# The oncogenic human B-cell lymphoma MYD88 L265P mutation genocopies activation by phosphorylation at the Toll/interleukin-1 receptor (TIR) domain

**DOI:** 10.1038/s41408-023-00896-6

**Published:** 2023-08-18

**Authors:** Marthe Minderman, Hildo Lantermans, Carmen van der Zwaan, Arie J. Hoogendijk, Maartje van den Biggelaar, Marie José Kersten, Marcel Spaargaren, Steven T. Pals

**Affiliations:** 1 Department of Pathology, Cancer Center Amsterdam, Amsterdam UMC, Location University of Amsterdam, Amsterdam, The Netherlands; 2 Lymphoma and Myeloma Center Amsterdam – LYMMCARE, Amsterdam, The Netherlands; 3 Department of Molecular Hematology, Sanquin Research, Amsterdam, The Netherlands; 4 Department of Hematology, Cancer Center Amsterdam, Amsterdam UMC, Location University of Amsterdam, Amsterdam, The Netherlands

**Keywords:** B-cell lymphoma, Cell signalling

## Abstract

MYD88 is the key signaling adaptor-protein for Toll-like and interleukin-1 receptors. A somatic L265P mutation within the Toll/interleukin-1 receptor (TIR) domain of MYD88 is found in 90% of Waldenström macroglobulinemia cases and in a significant subset of diffuse large B-cell lymphomas. MYD88-L265P strongly promotes NF-κB pathway activation, JAK-STAT signaling and lymphoma cell survival. Previous studies have identified other residues of the TIR-domain crucially involved in NF-κB activation, including serine 257 (S257), indicating a potentially important physiological role in the regulation of MYD88 activation. Here, we demonstrate that MYD88 S257 is phosphorylated in B-cell lymphoma cells and that this phosphorylation is required for optimal TLR-induced NF-κB activation. Furthermore, we demonstrate that a phosphomimetic MYD88-S257D mutant promotes MYD88 aggregation, IRAK1 phosphorylation, NF-κB activation and cell growth to a similar extent as the oncogenic L265P mutant. Lastly, we show that expression of MYD88-S257D can rescue cell growth upon silencing of endogenous MYD88-L265P expression in lymphoma cells addicted to oncogenic MYD88 signaling. Our data suggest that the L265P mutation promotes TIR domain homodimerization and NF-κB activation by copying the effect of MY88 phosphorylation at S257, thus providing novel insights into the molecular mechanism underlying the oncogenic activity of MYD88-L265P in B-cell malignancies.

## Introduction

B cells can detect antigen-specific signals through their B-cell antigen receptor (BCR) and damage- or pathogen-associated signals by the expression of Toll-like receptors (TLRs). Myeloid differentiation primary-response gene 88 (MYD88) is the key signaling adaptor molecule for both interleukin-1 receptors (IL-1Rs) and TLR-derived signals. Activating *MYD88* mutations are found in 10–20% of activated B-cell-like diffuse large B-cell lymphoma (ABC-DLBCL) cases, but are rare in germinal center-like DLBCL (GCB-DLBCL) and most other B cell non-Hodgkin lymphomas [1]. Remarkably, *MYD88* mutations are present in ~70% of primary testis lymphomas (PTLs), primary central nervous system lymphomas (PCNSLs) and primary vitreoretinal lymphomas (PVRLs) and in over 90% of Waldenström macroglobulinemia (WM) cases [2–7]. The vast majority of *MYD88* mutations involve the same amino acid substitution, i.e., a leucine to proline at position 265 (L265P), within the MYD88 Toll/interleukin-1 receptor (TIR) domain. This mutation strongly enhances NF-κB and JAK–STAT3 signaling and thereby promotes the survival of these lymphoma cells [1]. In DLBCL but not in WM, the *MYD88* L265P mutation frequently co-occurs with mutations in *CD79B*, an essential BCR complex-associated protein, suggesting that BCR and TLR signaling cooperate to optimally promote NF-kB signaling and tumor cell survival [2, 3, 8].

The regulation of TLR/MYD88 signaling in lymphomas without *MYD88* mutations, as well as in healthy B cells, is incompletely understood. Using mutagenesis assays in HEK293T cells, Vyncke et al. identified MYD88 residues essential for MYD88 TIR domain interactions and NF-κB activation. Interestingly, they demonstrated that phosphomimetic S255D and S257D mutations, which mimic physiological phosphorylation at these positions, have opposing effects on NF-κB activation: whereas the S257D mutant promotes hyperactivation of NF-κB, the S255D mutant has an inhibitory effect [9]. In line with these findings, previous studies have shown that an alanine substitution at position S257, but not at S255, impairs MYD88 TIR domain interactions and downstream NF-κB activation [10]. Importantly, both serine 255 and 257 were shown to be phosphorylated in TLR4-expressing HEK293T cells [11].

The above findings indicate a potentially important physiological role for serine 255 and 257 in the regulation of MYD88 activation, a role that might greatly impact B cell biology and lymphomagenesis. In support of this hypothesis, molecular dynamics simulation revealed that the phosphomimetic MYD88 S257D and the oncogenic L265P mutations provoke a similar conformational change in the MYD88 TIR domain [9]. These findings prompted us to directly explore the presence and functional consequences of MYD88 S257 phosphorylation in the cells of interest*,* i.e., in B lymphoma cells. In the current study, we demonstrate that MYD88 S257 indeed is phosphorylated in B lymphoma cells and that cells expressing a S257A mutant, impeding phosphorylation, display reduced NF-κB activation upon stimulation with TLR ligands. In addition, we demonstrate that the MYD88 S257D mutant, which mimics constitutive S257 phosphorylation, induces strong MYD88 aggregation, IRAK1 phosphorylation, NF- κB activity and proliferation in DLBCL cells, similar to the L265P mutant, and, moreover, rescues lymphoma cells addicted to MYD88 L265P upon silencing of this oncogene.

## Materials and methods

### Cell lines

OCI-LY1 and OCI-LY7 were cultured in IMDM (Thermo Fisher Scientific, Waltham, Massachusetts, USA) supplemented with 10% FCS. U2932 and RIVA were cultured in RPMI-1640 (Thermo Fisher Scientific) supplemented with 10% FCS and HBL-1 and TMD8 were cultured in RPMI-1640 supplemented with 20% FCS. OCI-LY10 was cultured in IMDM supplemented with 20% human serum (Sigma Aldrich, Saint Louis, Missouri, USA). OCI-LY1 and OCI-LY7 were kindly provided by Dr. U. Klein (University of Leeds, Leeds, United Kingdom). OCI-LY10, RIVA and TMD8 were kindly provided by Dr. G. Lenz (University Hospital Münster, Münster, Germany). All cell lines were frequently tested for mycoplasma contamination using RT-qPCR and cell line authentication was routinely performed using Short Tandem Repeat DNA profiling (PowerPlex 16, Promega, Madison, Wisconsin, USA).

### Cloning, transfection and transduction

The MYD88 coding sequence was subcloned into LZRS-IRES-GFP (Addgene plasmid #21961). Subsequently, the QuikChange II Site-Directed Mutagenesis Kit (Agilent, Santa Clara, California, USA) was utilized to generate the MYD88 mutant constructs according to the manufacturer’s instructions. Mutants were confirmed by Sanger sequencing. To generate doxycycline-inducible MYD88 knockdown cell lines, we inserted an shRNA targeting MYD88 (GCAGAGCAAGGAATGTGACTT or GACCCAATGTACCAGTATT) into Tet-pLKO-puro (Addgene plasmid #21915). To generate MYD88 knockout cells, we inserted a single guide RNA targeting MYD88 (CTGCTCTCAACATGCGAGTG or CTCGAGCAGTCGGCCTACAG) into pL-CRISPR.EFS.GFP (Addgene plasmid #57818). For generation of stable Cas9 expressing cell lines, we used lentiCas9-Blast (Addgene plasmid #52962). MSCV-CA-IKK2-IRES-GFP was kindly provided by Dr J. Schuringa (University of Groningen, Groningen, The Netherlands).

### RNA isolation, cDNA synthesis and RT-qPCR

Total RNA was extracted using TRI-reagent according to the manufacturer’s instructions (Sigma Aldrich) and converted to cDNA using oligo(dT) primers for 1 h at 37 °C. RT-qPCRs were executed using Sensifast (Bioline, London, UK) on a on a Lightcycler 480 (Roche, Basel, Switzerland). Expression levels were normalized to expression of *RPLP0*. Primers used were: MYD88 fw (5′– GAGGCTGAGAAGCCTTTACAGG –3′); MYD88 rv (5′– GCAGATGAAGGCATCGAAACGC –3′); HCK fw (5′– TGGCAGTGAAGACGATGAAG –3′); HCK rv (5′– GTAGATGGGCTCCTTGGTGA –3′); CD80 fw (5′– TAGATGCGAGTTTGTGCCAG –3′); CD80 rv (5′– GCTGGCTGGTCTTTCTCACT–3′); RPLP0 fw (5′– GCTTCCTGGAGGGTGTCCGC –3′); RPLP0 rv (5′– TCCGTCTCCACAGACAAGGCCA–3′).

### Flow cytometry and viability assays

Cells were fixed with 4% PFA and subsequently permeabilized with permeabilization buffer (PBS, 1% BSA, 0.1% Saponin) for 15 min on ice. Subsequently, cells were incubated with anti-phospho-IRAK1 (Thr209) antibody for 30 min on ice (ab218130, Abcam, Cambridge, UK) followed by goat anti-rabbit IgG -DyLight® 650 (ab96886, Abcam) for another 30 min. For cell surface staining, cells were incubated with anti-CD80-PE (clone 2D10.4, eBioscience, San Diego, California, USA) for 30 min at 4 degrees.

Cell viability was assessed using 7-AAD viability staining solution (Thermo Fisher Scientific). For Annexin-V viability assays [12], cells were incubated with anti-Annexin V- APC (BioLegend, San Diego, California) for 30 min at 4 degrees followed by 5 min with propidium iodide (PI) (Thermo Fisher Scientific), both diluted in Annexin-V binding buffer (0.1 M Hepes (pH 7.4), 1.4 M NaCl, and 25 mM CaCl2). To assess proliferation, cells were stained with eFluor 660 dye according to manufacturer’s instructions (Thermo Fisher Scientific). All flow cytometric measurements were performed on a FACSCanto II flow cytometer (BD Biosciences, San Jose, California, USA).

### Immunoblotting

Cells were lysed in RIPA buffer (20 mM Tris-HCl pH 7.4, 150 mM NaCl, 1% NP-40, 0.5% Sodium deoxycholate, 0.1% SDS, 5 mM EDTA, 10% glycerol). Primary antibodies used were: mouse anti-β-tubulin (clone D66, Sigma Aldrich), mouse anti-β-actin (clone AC-15, Sigma-Aldrich), rabbit anti-MYD88 (clone D80F5, Cell Signaling Technology, Danvers, Massachusetts, USA), rabbit anti-HCK (clone E1I7F, Cell Signaling Technology), rabbit anti-phospho-NF-κB p65 (Ser536) (clone 93H1, Cell Signaling Technology) and mouse anti-p65 (clone F-6, Santa Cruz Biotechnology). Secondary antibodies used were anti-mouse-HRP and anti-rabbit-HRP (both from DAKO, Santa Clara, California, USA). Images were acquired on a ImageQuant LAS 4000 (GE Healthcare Life Sciences) and quantification of immunoblot membranes was performed using Image Lab software (Bio-Rad Laboratories, Hercules, California, USA).

### Statistical analysis

Data are presented as mean ± SD of at least three independently performed experiments. Experimental data were analyzed using one-way ANOVA followed by Tukey’s multiple comparisons test or two-way ANOVA followed by Sidak’s multiple comparisons test. The differences were considered significant when *p* < 0.05.

For further details and other methods, see “Supplemental Methods”

## Results

### MYD88 TIR domain serine 257 mediates TLR-induced NF-kB activation in DLBCL cells

**Fig. 1:**
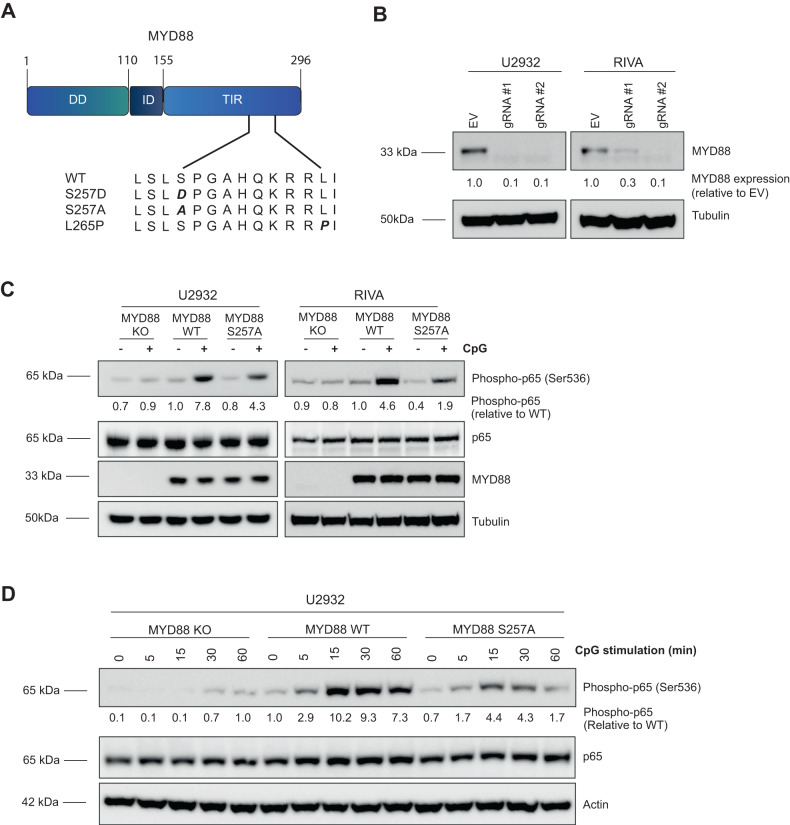
TLR-induced NF-κB activation is repressed in cells expressing the MYD88 S257A mutant. **A** A schematic diagram of wild-type and mutant MYD88 proteins. Amino acid positions are shown according to protein accession NCBI NP_002459.2. **B** Immunoblot analysis of MYD88 in U2932 and RIVA transduced lentiCas9-Blast and subsequently transfected with pLentiGuide-GFP containing two different single guide RNAs (sgRNA) targeting MYD88. Cells were allowed to recover for 48 h after transfection before the GFP positive cells were sorted. β-tubulin was used as loading control. **C** Immunoblot analysis of phosphorylated p65 (Ser536) and MYD88 in U2932 and RIVA expressing MYD88 WT or MYD88 S257A. Cells were serum starved for 1 h at 37 °C before stimulation for 15 min with 500 ng/ml CpG. Total p65 and β-tubulin were used as loading controls. **D** Immunoblot analysis of phosphorylated p65 (Ser536) and MYD88 in U2932 expressing MYD88 WT or MYD88 S257A. Cells were serum starved for 1 h at 37 °C before stimulation for 5, 15, 30 or 60 min with 500 ng/ml CpG. Total p65, β-actin and β-tubulin were used as loading controls.

Phosphorylation of MYD88 at S257 has been previously described in various cell line models, including HEK293T cells, HeLa cells and K562 leukemia cells [11, 13–15]. In B cells, however, phosphorylation has not yet been reported. By employing label-free quantitative mass spectrometry (MS), we detected MYD88 S257 phosphorylation in the DLBCL cell line U2932, which contains unmutated (wildtype) MYD88 (Supplemental Fig. 1A). Please note that, to align with the annotation of the oncogenic MYD88 L265P mutant first described by Ngo et al., amino acid positions in the current study are shown according to protein accession NCBI NP_002459.2 (Fig. 1A). MYD88 S257 and L265 correspond to S244 and L252, respectively, annotated according to the canonical sequence (NP_002459.3).

To establish whether MYD88 phosphorylation at S257 is involved in TLR-mediated NF-κB signaling in B lymphoma, we generated an alanine mutant at position 257 (S257A), disabling phosphorylation. Subsequently, either this S257A mutant or wildtype MYD88 was introduced in the ABC DLBCL cell lines U2932 and RIVA in which the endogenous (wild type) MYD88 protein had been deleted using CRISPR technology (Fig. 1B), to rule out signaling via endogenous MYD88. TLR-induced NF-κB activation was effectively abrogated in MYD88 KO U2932 and RIVA cells, confirming functional knockout (Fig. 1C, Fig. 1D and Supplemental Fig. 1B). Equal levels of ectopically expressed wildtype and mutant MYD88 S257A were confirmed by immunoblotting (Fig. 1C and Supplemental Fig. 1B). Interestingly, TLR-induced NF-κB activation was substantially repressed in cells expressing the MYD88 S257A mutant compared to wildtype MYD88. These results were observed for stimulation with the TLR9 ligand CpG (Fig. 1C and Fig. 1D) as well as for the TLR1/2 ligand Pam3CSK4 (Supplemental Fig. 1B). Both U2932 and RIVA showed a stronger response to CpG stimulation as compared to stimulation with Pam3CSK4. The reduction in NF-κB activation in MYD88 S257A expressing cells was observed after 15, 30 and 60 min of TLR stimulation (Fig. 1D). These findings indicate that MYD88 S257 phosphorylation is required for optimal TLR-mediated NF-κB activation.

### The phosphomimetic MYD88 S257D and oncogenic MYD88 L265P mutants both promote molecular aggregation of MYD88

**Fig. 2:**
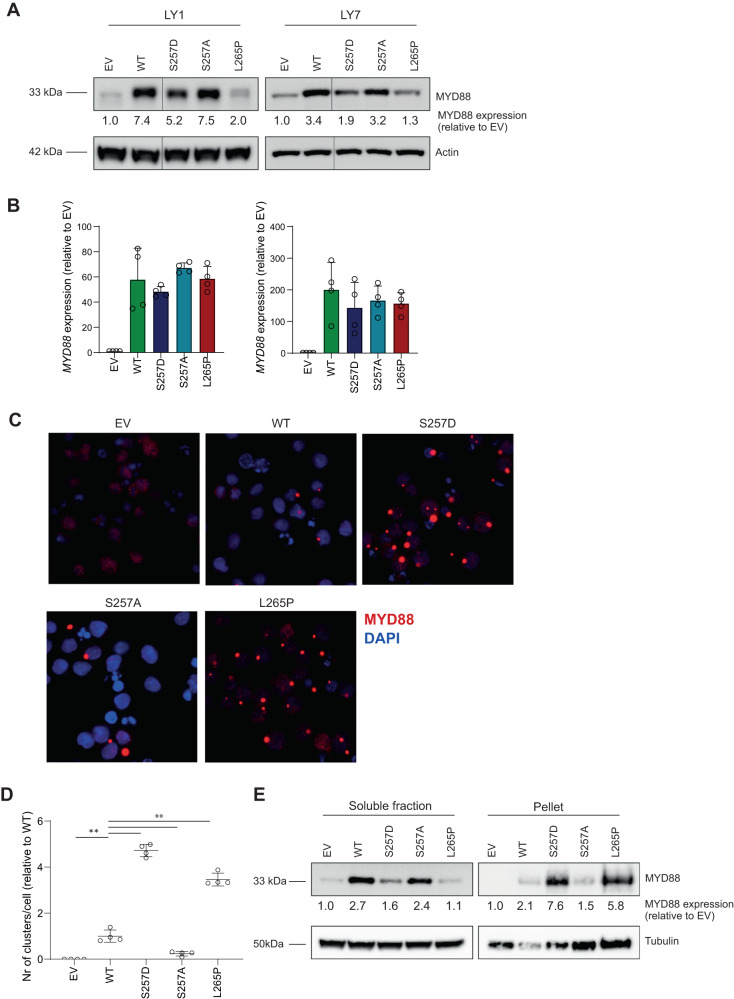
Expression of MYD88 S257D and L265P induces molecular aggregation. **A** Immunoblot analysis for MYD88 in OCI-LY1 and OCI-LY7 transduced with different MYD88 mutants. Cells were transduced with an empty vector (EV) or an expression vector for MYD88 (WT, S257D, S257A or L265P) and sorted for GFP expression. β-actin was used as loading control. **B** RT-qPCR analysis of *MYD88* expression in OCI-LY1 and OCI-LY7 transduced with different MYD88 mutants. Cells were allowed to recover for 72 h before RNA isolation. *RPLP0* was used as an input control and data are normalized to the EV control expression levels. The mean ± SD of four independent experiments performed in triplicate is shown. **C** Immunofluorescence analysis of MYD88 in OCI-LY7 cells. Cells were fixed and stained with an anti-MYD88 antibody followed by goat-anti-mouse IgG-AF594 antibody (red) and nuclei were counterstained with DAPI (blue). **D** Quantification of the number of clusters per cell determined using ImageJ software. Values are normalized to the number of clusters per cell for cells expressing WT MYD88. The mean ± SD of four independent experiments performed is shown. **E** Immunoblot analysis comparing MYD88 expression in the soluble fraction versus the pellet fraction in OCI-LY7 transduced with different MYD88 mutants. Cell lysates were fractionated by centrifugation. β-tubulin was used as loading control.

To further elucidate the role of serine phosphorylation in MYD88 signaling, we generated expression constructs encoding the activating MYD88 S257D phosphomimetic mutant and the oncogenic L265P mutant (Fig. 1A). Next, we retrovirally introduced all MYD88 variants in DLBCL cell lines OCI-LY1 and OCI-LY7, which endogenously express wildtype MYD88 and display very weak basal NF-κB activation. Immunoblot analysis shows that we achieved increased MYD88 expression levels, compared to empty vector (EV) transduced cells (Fig. 2A). Surprisingly, protein expression of the phosphomimetic MYD88 S257D and oncogenic MYD88 L265P was remarkably lower as compared to wildtype MYD88. RT-PCR analysis shows that these differences in protein level were not caused by differential expression at the transcriptional level (Fig. 2B).

Interestingly, using confocal microscopy Avbelj et al. previously demonstrated that MYD88 L265P, as well as other lymphoma-associated mutants, strongly promote aggregation of MYD88 [16]. Moreover, these authors established that this aggregation renders the MYD88 protein less soluble, causing it to end up in the pellet fraction of a protein lysate. Using immunofluorescence (IF) microscopy, we confirmed that MYD88 L265P indeed strongly promotes aggregation of MYD88 (Fig. 2C and D). Interestingly, the phosphomimetic MYD88 S257D mutant similarly showed strong aggregation, while aggregation was hardly observed for wildtype MYD88 or MYD88 S257A. Using immunoblot analysis, we demonstrated that MYD88 S257D and L265P levels were significantly enriched in the insoluble/pellet fraction, further supporting the presence of molecular aggregation (Fig. 2E).

### The phosphomimetic MYD88 S257D is equally potent as the MYD88 L265P oncogenic mutant in NF-κB pathway activation

To assess whether the MYD88 aggregation caused by MYD88 S257D or L265P expression, correlates to enhanced signaling, we studied TLR/MYD88/NF-κB pathway activation. Ligand recognition by TLRs promotes the recruitment of MYD88 to the TIR domain of TLRs. This triggers recruitment of IRAK4 and IRAK1 to the receptor complex via their respective death-domains (DD) [17]. The subsequent activation of IRAK1 is a multistep process, which first requires phosphorylation of threonine 209, resulting in a conformational change of the kinase domain, allowing further phosphorylation steps to occur [18].

**Fig. 3:**
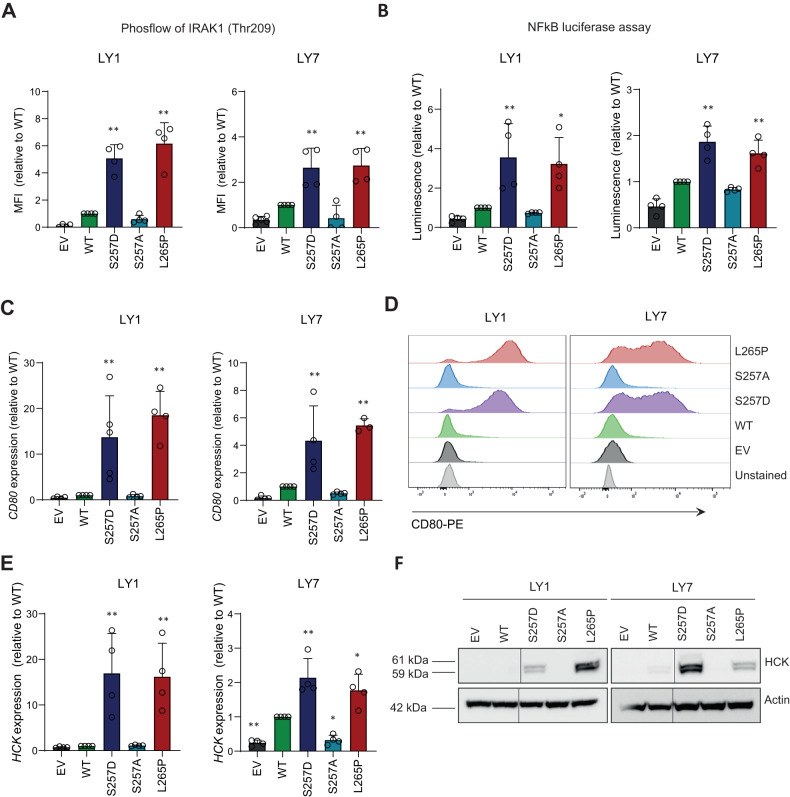
Expression of MYD88 S257D and L265P promote NF-kB activation. **A** Phosflow analysis for IRAK1(Threonine 209) in OCI-LY1 and OCI-LY7 transduced with different MYD88 mutants. Cells were transduced with an empty vector (EV) or an expression vector for MYD88 (WT, S257D, S257A or L265P) and sorted for GFP expression. Untransduced, GFP negative, cells were used as negative control. IRAK1(Thr209) phosphorylation levels are shown relative to cells expressing WT MYD88. The mean ± SD of four independent experiments is shown. **P < 0.01 using 1-way ANOVA with Dunnett’s multiple comparisons test. **B** NF-kB activity measured by NF-kB-dependent luciferase reporter assays in OCI-LY1 and OCI-LY7 transduced with different MYD88 mutants. Cells were stably transduced with different MYD88 mutants and subsequently transfected with plasmid DNAs encoding for NF-κB promoter-driven firefly luciferase and a Renilla luciferase as control. Cells were allowed to recover for 48 h after transfection. Firefly luciferase activity was first normalized to the activity of Renilla luciferase. Then, luciferase activity was normalized to cells expressing WT MYD88. The mean ± SD of four independent experiments performed is shown. *P < 0.05; **P < 0.01 using 1-way ANOVA with Dunnett’s multiple comparisons test. **C**, **E** RT-qPCR analysis of *CD80* (**C**) or *HCK* (**E**) expression in OCI-LY1 and OCI-LY7 transduced with different MYD88 mutants. Cells were allowed to recover for 72 h before RNA isolation. *RPLP0* was used as an input control and data are normalized to the EV control expression levels. The mean ± SD of four independent experiments performed in triplicate is shown. **P < 0.01 using 1-way ANOVA with Dunnett’s multiple comparisons test. **D** Flow cytometric analysis showing membranous CD80 staining in OCI-LY1 and OCI-LY7 transduced with different MYD88 mutants. One representative experiment of three independent experiments is shown. **F** Immunoblot analysis for HCK in OCI-LY1 and OCI-LY7 transduced with different MYD88 mutants. β-actin was used as loading control.

By employing phosflow analysis, we established that both MYD88 S257D and MYD88 L265P promote IRAK1 phosphorylation at threonine 209 in both OCI-LY1 and OCI-LY7 (Fig. 3A). Expression of wildtype MYD88 and MYD88 S257A resulted in a small increase in IRAK1 phosphorylation compared to empty vector (EV) transduced cells. To confirm that increased IRAK1 phosphorylation indeed results in enhanced downstream NF-κB pathway activation, we performed NF-κB luciferase assays. These assays demonstrated that both MYD88 S257D and L265P strongly promote NF-κB pathway activation in both DLBCL cell lines (Fig. 3B). Introduction of both wildtype MYD88 and MYD88 S257A weakly stimulated NF-κB pathway activation compared to cells expressing only endogenous MYD88. In addition, MYD88 S257D and L265P greatly enhanced the expression of the NF-κB target genes *CD80* and *HCK* (Fig. 3C, E). A similar upregulation of *CD80* and *HCK* was observed upon expression of constitutively active IKK-β (CA-IKK2) (Supplemental Fig. 2A). Using cell surface staining for CD80 and immunoblot analysis for HCK, we confirmed that the changes in mRNA transcription resulted in enhanced protein expression levels (Fig. 3D, F). Together, these data demonstrate that the phosphomimetic MYD88 S257D mutant is a potent mediator of NF-κB pathway activation in B cells with an efficacy similar to that of the oncogenic MYD88 L265P mutant.

### Phosphomimetic MYD88 S257D and oncogenic MYD88 L265P similarly promote proliferation of MYD88 wildtype DLBCL cells

**Fig. 4:**
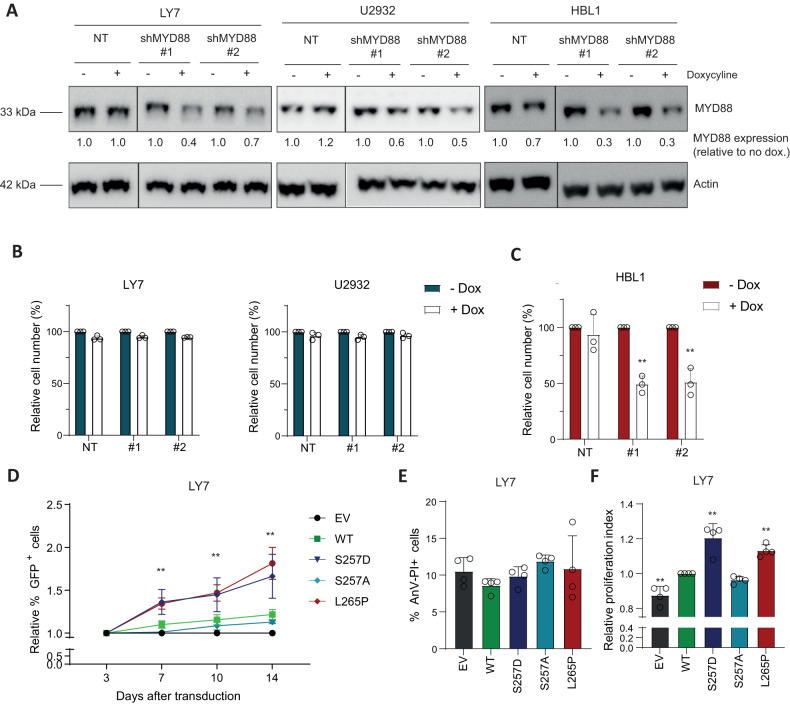
MYD88 257D and L265P augment cell growth in MYD88 wildtype cells. **A** Immunoblot analysis of MYD88 in OCI-LY7, U2932 and HBL-1 treated for 3 days with 500 ng/ml doxycyline. β-actin was used as loading control. **B**, **C** Flow cytometric analysis in OCI-LY7 and U2932 (**B**) and HBL-1 (**C**) of the number of viable cells, as determined by 7-AAD staining, after 5 days of treatment with 500 ng/ml doxycycline in cell lines transduced with inducible shRNA constructs targeting *MYD88*. The number of viable cells was normalized to the vehicle-treated condition. Data are presented as mean ± SD of four independent experiments. **P < 0.01 using 2-way ANOVA with Bonferroni’s multiple comparisons test. **D** Flow cytometric analysis of OCI-LY7 cells transduced with an empty vector (EV) or an expression vector for MYD88 (WT, S257D, S257A or L265P) co-expressing GFP. The percentage of GFP positive cells was followed in time and plotted as the percentage of GFP^+^ cells, normalized to the value at day 3 following retroviral transduction. The mean ± SD of three independent transductions is shown. **E** Flow cytometric analysis showing the percentage of cells double positive for Annexin-V and Propidium Iodide (PI) staining. OCI-LY7 cells were transduced with different MYD88 mutants or a EV control. Data are presented as mean ± SD of four independent experiments. **F** Flow cytometric analysis showing the relative proliferative index of OCI-LY7 cells transduced with different MYD88 mutants or a EV control. Cells were stained with cell proliferation dye eFluor 660 and analyzed 3 days after staining. Values are normalized to the proliferative index of cells expressing WT MYD88. Data are presented as mean ± SD of four independent experiments. **P < 0.01 using 1-way ANOVA with Dunnett’s multiple comparisons test.

Since NF-κB signaling is crucial in mediating survival and proliferation of B cells, we next set out to explore the contribution of MYD88 S257D and L265P to cell growth and viability. In contrast to cells expressing wildtype MYD88, ABC DLBCL and WM cells bearing the L265P mutation have been shown to critically depend on MYD88 for their survival [1, 19]. We corroborated these findings in the MYD88 WT cell lines OCI-LY7 and U2932 and the MYD88 L265P expressing cell line HBL-1, using inducible shRNA-mediated silencing of MYD88. Efficient knockdown of MYD88 upon doxycycline treatment was confirmed by immunoblot analysis (Fig. 4A). In line with previous studies [1], silencing of MYD88 did not affect cell growth in MYD88 WT cell lines OCI-LY7 and U2932 (Fig. 4B), but strongly reduced cell growth in MYD88 L265P expressing cell line HBL-1 (Fig. 4C).

Next, we assessed the effect of the different MYD88 variants on the proliferation and viability of MYD88 WT cells. To this end, we transduced OCI-LY1 and OCI-LY7 with a bicistronic vector co-expressing MYD88 and GFP. In OCI-LY7 both MYD88 S257D and MYD88 L265P expressing cells progressively outcompeted their untransduced counterparts, indicating that these cells have a competitive growth advantage (Fig. 4D). By contrast, the percentage of cells transduced with an empty vector (EV) or MYD88 S257A was stable over time, while the percentage of cells ectopically expressing wildtype MYD88 showed a small, non-significant, increase. Notably, the growth advantage upon expression of MYD88 S257D and L265P was similar to that observed upon expression of CA-IKK2 (Supplemental Fig. 2B). Surprisingly, in OCI-LY1 expression of MYD88 S257D and L265P, but also of CA-IKK2, did not confer a growth advantage to the cells (Supplemental Fig. 2B and C).

Subsequently, we explored whether the growth advantage in OCI-LY7 could be attributed to increased cell survival or to enhanced cell proliferation. For this purpose, we performed Annexin-V-PI stainings and utilized a proliferation dye to track cell divisions. We observed ~10% Annexin-V-PI positive cells in EV control cells, this percentage was not significantly altered by any of the MYD88 mutants (Fig. 4E). However, introduction of either *MYD88* S257D or *MYD88* L265P resulted in a significant increase in cell proliferation compared to expression of wildtype *MYD88* (Fig. 4F). These findings indicate that MYD88 S257D and L265P provide a growth advantage, not by affecting cell viability, but through increased proliferation.

### Phosphomimetic MYD88 S257D rescues growth of DLBCL cells addicted to the MYD88 L265P oncoprotein

Following silencing of endogenous MYD88 in L265P-expressing lymphoma cells lines, cell growth could only be sustained by mutant, but not wildtype MYD88, demonstrating that L265P is a gain-of-function mutation [1]. To explore whether the phosphomimetic MYD88 S257D is also capable of rescuing cell growth, we performed a complementation experiment: We knocked-down endogenous mutant MYD88 using an shRNA targeting the *MYD88* 3′UTR (shRNA #2) and stably expressed either wildtype or mutant MYD88 coding domains. Because of a low retroviral transduction efficiency, the ABC DLBCL cell line HBL-1 (previously used in Fig. 4) could not be used in these experiments.

**Fig. 5:**
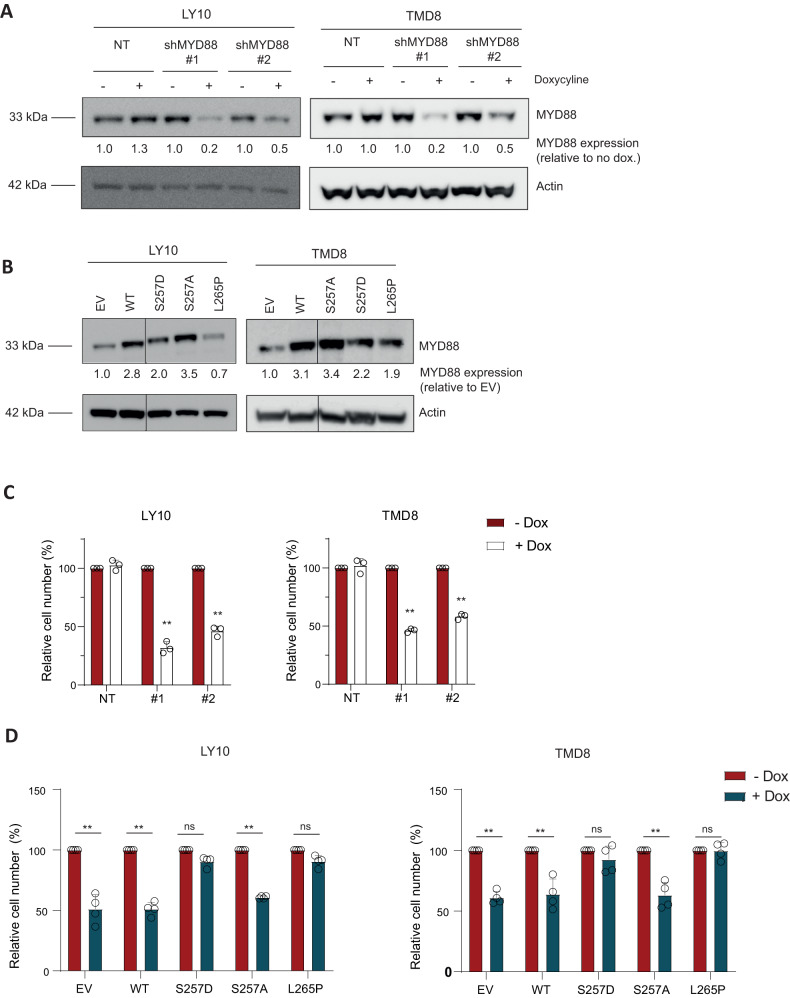
MYD88 S257D rescues cell growth in MYD88 L265P addicted cell lines. **A** Immunoblot analysis of MYD88 in OCI-LY10 and TMD8 treated for 3 days with 500 ng/ml doxycyline. β-actin was used as loading control. **B** Immunoblot analysis for MYD88 in OCI-LY10 and TMD8 transduced with different MYD88 mutants. Cells were transduced with an empty vector (EV) or an expression vector for MYD88 (WT, S257D, S257A or L265P) and sorted for GFP expression. β-actin was used as loading control. **C** Flow cytometric analysis in OCI-LY10 and TMD8 of the number of viable cells, as determined by 7-AAD staining, after 5 days of treatment with 500 ng/ml doxycycline in cell lines transduced with inducible shRNA constructs targeting *MYD88*. The number of viable cells was normalized to the vehicle-treated condition. Data are presented as mean ± SD of three independent experiments. **P < 0.01 using 2-way ANOVA with Bonferroni’s multiple comparisons test. **D** Flow cytometric analysis of the number of viable cells after 5 days of treatment with 500 ng/ml doxycycline in cell lines OCI-LY10 or TMD8 transduced with an inducible shRNA construct targeting *MYD88 3’UTR* in combination with an empty vector (EV) or an expression vector for MYD88 (WT, S257D, S257A or L265P). The number of viable cells was normalized to the vehicle-treated condition. Data are presented as mean ± SD of four independent experiments. **P < 0.01 using 2-way ANOVA with Bonferroni’s multiple comparisons test.

Efficient knockdown of MYD88 upon doxycycline treatment was confirmed by immunoblot analysis (Fig. 5A). Consistent with the molecular aggregation of MYD88 previously observed in OCI-LY7, protein levels of MYD88 S257D and L265P expressed in OCI-LY10 and TMD8 were lower compared to those in wildtype MYD88 and MYD88 S257A (Fig. 5B). In line with previous research [1], inducible shRNA-mediated silencing of MYD88 resulted in a significant reduction in the number of viable cells in the MYD88 L265P expressing cell lines OCI-LY10 and TMD8 (Fig. 5C); introduction of MYD88 L265P, but not wildtype MYD88, annulled this effect (Fig. 5D). Importantly, however, ectopic expression of phosphomimetic MYD88 S257D, but not S257A, similarly offset the effect of silencing of endogenous oncogenic MYD88 L265P (Fig. 5D). In the presence of endogenous MYD88 L265P, expression of MYD88 S257A did not affect cell survival (Supplemental Fig. 3). This implies that MYD88 S257A does not function as a dominant negative mutant and that MYD88 L265P can still activate the TLR-MYD88-NFKB pathway in these cells. Taken together, our findings show that phosphomimetic MYD88 S257D and oncogenic L265P are equally able to propagate cell growth in DLBCL cells addicted to oncogenic MYD88 signaling, supporting the hypothesis that the oncogenic MYD88 L265P mutation exerts its oncogenic function by mimicking the activating effect of MYD88 serine 257 phosphorylation.

## Discussion

The MYD88 TIR domain plays an essential role in the interactions with TLR/IL1-Rs as well as in MYD88 homodimerization [20]. Upon dimerization, MYD88 recruits the downstream kinases IRAK1 and IRAK4 via their respective death-domains to form a large multiprotein complex called the Myddosome. Phosphorylated IRAK1 dissociates from the receptor complex to interact with the E3 ubiquitin ligase TRAF6, to finally activate the MAPK and NF-kB signaling pathways. In 2011, Ngo et al. first described the MYD88 L265P mutation in the hydrophobic core of the MYD88 TIR domain as oncogenic driver in DLBCL [1]. Subsequent studies have identified residues of the TIR domain that are crucially involved in MYD88 homodimerization and NF-κB activation, in particular serine 257 [9, 10]. In the current study, we show that MYD88 S257 phosphorylation is required for optimal NF-κB activation in DLBCL and that a phosphomimetic (S257D) mutant promotes IRAK1 phosphorylation, NF-κB activity, and cell growth with a similar efficacy as the oncogenic MYD88 L265P mutant and, moreover, can rescue DLBCL cells addicted to this oncogenic driver.

We demonstrate that MYD88 can be phosphorylated at S257 in DLBCL cells (Supplemental Fig. 1A). Moreover, we show that substitution of this serine residue by alanine (S257A) strongly reduces TLR-induced NF-κB activation in DLBCL (Fig. 1). These data confirm and extend previous studies showing MYD88 S257 phosphorylation in macrophages and TLR4 stable HEK293T cells and establish its importance for MYD88-dependent NF-κB activation in B lymphoma cells [10, 11].

Avbelj et al. previously proposed that MYD88 L265P, as well as other lymphoma-associated MYD88 mutations, are prone to spontaneously oligomerize and form Myddosome complexes [16]. Using molecular dynamics simulation, these authors demonstrated that mutated residues of MYD88 L265P and other lymphoma-associated mutants are shielded and likely affect interacting residues through an allosteric effect [16]. Several studies have indeed established that MYD88 L265P causes increased homodimerization of the MYD88 TIR domain leading to constitutive activity [9, 21, 22]. Our current data confirm that the oncogenic MYD88 L265P mutant induces strong MYD88 aggregation, IRAK1 phosphorylation and NF-κB activation in DLBCL cells (Fig. 2 and Fig. 3). Interestingly, we moreover demonstrate that the phosphomimetic MYD88 S257D promotes aggregation, phosphorylation, and NF-κB activation in DLBCL with a similar efficacy, hence mimicking the functional effects of the oncogenic mutant (Fig. 2 and Fig. 3). In line with these findings, studies in a cell-free system and in HEK293T cells showed that the phosphomimetic S257D mutant had a higher propensity to oligomerize as compared to wildtype MYD88 [23] and induces strong induction of NF-κB reporter activity [9, 24]. Inversely, HEK293T cells expressing a S257A mutant, which impedes phosphorylation, displayed impaired MYD88 homodimerization, interaction with IRAKs and activation of NF-κB signaling [10].

Importantly, we found that the phosphomimetic MYD88 S257D and oncogenic L265P mutant were equally capable of promoting lymphoma cell growth (Fig. 4). MYD88 S257D did not only promote cell growth of MYD88 wildtype cells, but, remarkably, was also able to sustain cell growth of MYD88 L265P-addicted lymphoma cells after silencing of endogenous (mutant) MYD88 (Fig. 5). Based on molecular dynamics simulation studies, Vyncke *et al*. suggested that MYD88 S257D and L265P preferentially adopt the same conformation of the CD loop, which promotes MYD88 TIR-TIR domain interactions [9]. Indeed, our observations strongly support the hypothesis that MYD88 L265P exerts its oncogenic functions by mimicking the functional and conformational effects of MYD88 serine 257 phosphorylation.

Interestingly, Xie et al. reported that the S257 residue of MYD88 can be dephosphorylated by protein phosphatase 2 A catalytic subunit α (PP2Ac) [11]: Constitutively activated PP2Ac dephosphorylated MYD88, resulting in reduced TLR-MYD88 interactions and, consequently, suppressed MYD88-NFκB-dependent gene transcription [11]. In line with these findings, in LPS-stimulated myeloid cells, knockout of PP2Ac resulted in increased NF-κB activity and enhanced secretion of pro-inflammatory cytokines [24]. To date, the kinase(s) responsible for MYD88 S257 phosphorylation remain unknown. Since MYD88 L265P was shown to associate with TLR9 and the BCR in the so-called ‘My-T-BCR’ complex, it is tempting to speculate that MYD88 S257 phosphorylation might also play an essential part in the cooperation of TLR and BCR signaling [25]. Consistent with this notion, neural network–based kinase prediction (NetPhos 3.1) designates p38MAPK as putative candidate for MYD88 S257 phosphorylation [26]. Additional studies are required to unravel the signaling cascade and kinases involved in MYD88 S257 phosphorylation.

Better understanding of MYD88 TIR domain assembly has resulted in the development of peptidomimetic compounds that inhibit Myddosome formation [27, 28]. These compounds can provide an interesting therapeutic option for B-cell malignancies as well as for inflammatory diseases [29–32]. Our findings suggest that these compounds will not only be effective in MYD88 L265P-expressing lymphomas, but also in lymphomas in which MYD88 activation is regulated by other means, such as phosphorylation.

In conclusion, our findings support a model in which the oncogenic MYD88 L265P mutant drives lymphomagenesis by genocopying the conformational effects of physiological MYD88 S257 phosphorylation on TIR domain homodimerization, resulting in constitutive IRAK1 phosphorylation, NF-κB signaling and enhanced cell growth. Further studies are needed to identify the actors involved in the regulation of MYD88 TIR domain phosphorylation.

### Supplementary information

Supplemental Methods and Supplemental Figures

## Data Availability

The datasets generated during and/or analyzed during the current study are available from the corresponding author on reasonable request.
