# Supplementary material for: The oncogenic human B-cell lymphoma MYD88 L265P mutation genocopies activation by phosphorylation at the Toll/interleukin-1 receptor (TIR) domain
Source: Blood Cancer J. 2023 Aug 18;13(1):125. doi: 10.1038/s41408-023-00896-6 (PMC10435502; doi:10.1038/s41408-023-00896-6)
Supplement: Supplementary file 1 — Supplemental Methods and Supplemental Figures [file 41408_2023_896_MOESM1_ESM.pdf]

Supplemental Figures and Supplemental Methods

Supplemental methods

Immunofluorescence staining (IF)

Following fixation with 4% PFA, cells were permeabilized with permeabilization buffer (PBS, 1% BSA, 0.1% Saponin) for 30 minutes at RT. Slides were incubated o/n at 4°C with mouse anti-MYD88 (clone E-11, Santa Cruz, Dallas, Texas, USA). Then, slides were incubated 1 hour at RT with goat anti-mouse IgG - Alexa Fluor 594 (Thermo Fisher Scientific) followed by a counterstain with 0.5 µg/ml DAPI in PBS for 5 min at RT. Images were acquired using the automated Leica DM5000 B microscope and Leica Application Suite (LAS) software (Leica Microsystems, Wetzlar, Germany).

Luciferase assays

5 x 10^6 cells were transfected with 5 µg pNF-κB-Luc Cis-Reporter Plasmid (Agilent Technologies) and 0.1 µg pRL Renilla Luciferase Control Reporter Vector (Promega) using the Amaxa Nucleofector II in combination with program G16 and buffer T (Lonza, Basel, Switzerland). 48 hours following transfection, firefly and renilla luciferase activity were measured using the Dual-Glo® Luciferase Assay System according to manufacturer's instructions (Promega). Renilla luciferase activity served as a control for transfection efficiency.

Mass spectrometry

Tryptic digests were fractioned in using a High pH Reversed-Phase Peptide Fractionation kit (Pierce) according to manufacturer's instructions. Phosphopeptide enrichment was performed using Fe(III)-IMAC cartridges (Agilent) as described by Post *et al.* (13) on an AssayMAP BRAVO (Agilent). Peptides were separated by nanoscale C18 reverse chromatography coupled on-line to an Orbitrap Fusion Tribrid

mass spectrometer (Thermo Fisher Scientific) via a nanoelectrospray ion source at 2.15 kV. Buffer A was composed of 0.1% formic acid and buffer B of 0.1% formic acid and 80% acetonitrile. Peptides were loaded for 17 min at 300 nL/min at 5% buffer B, equilibrated for 5 min at 5% buffer B (17–22 min) and eluted by increasing buffer B from 5–27.5% (22–122 min) and 27.5–40% (122–132 min), followed by a 5 min wash to 95% and a 6 min regeneration to 5%. Survey scans of peptide precursors from 300 to 1600 m/z were performed at 120,000 resolution (at 200 m/z) with a 4×10^5 ion count target. Tandem mass spectrometry was performed by isolation with the quadrupole, with isolation window 1.6, higher energy collisional dissociation (HCD) fragmentation with normalized collision energy of 30 and rapid scan mass spectrometry analysis in the orbitrap. The tandem mass spectrometry (MS2) ion count target was set to 5×10^4, and the max injection time was 54 ms. Only those precursors with charge state 2–7 were sampled for MS2. The dynamic exclusion duration was set to 30 s with a 10 ppm tolerance around the selected precursor and its isotopes. Monoisotopic precursor selection was turned on. The instrument was run in top N mode. All data were acquired with Xcalibur software (Thermo Fisher Scientific). RAW mass spectrometry files were processed with the MaxQuant 2.0.1.0 computational platform. Proteins and peptides were identified using the Andromeda search engine by querying the human Uniprot database (release February 2019). Standard settings with the additional options match between runs and Phospho STY was set as dynamic modification.

Supplemental Figure 1

A

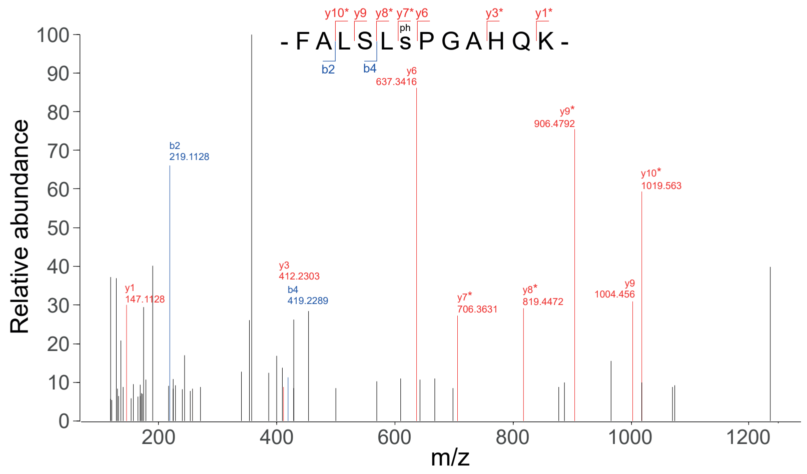

B

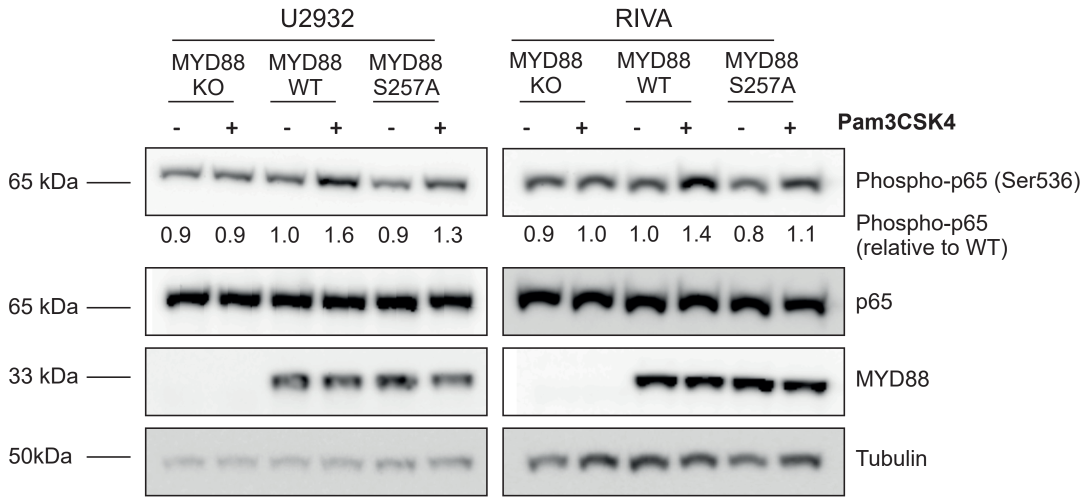

Supplemental Figure 1. (A) MaxQuant annotated MS/MS spectrum of the phosphorylated S257 MYD88 peptide FALSLs(phospho)PGAHQK. Each line on the x-axis represents an ion having a specific mass-to-charge ratio (m/z) and the height of the line indicates the relative abundance of the ion. b# indicate b-ion fragments with amino acid sequences in the peptide reading direction from left to right, y# ions from right to left. (B) Immunoblot analysis of phosphorylated p65 (Ser536) and MYD88 in U2932 and RIVA expressing MYD88 WT or MYD88 S257A. Cells were serum starved for 1 hour at 37ºC before stimulation for 15 minutes with 1 µg/ml Pam3CSK4. Total p65 and β-tubulin were used as loading controls.

Supplemental Figure 2

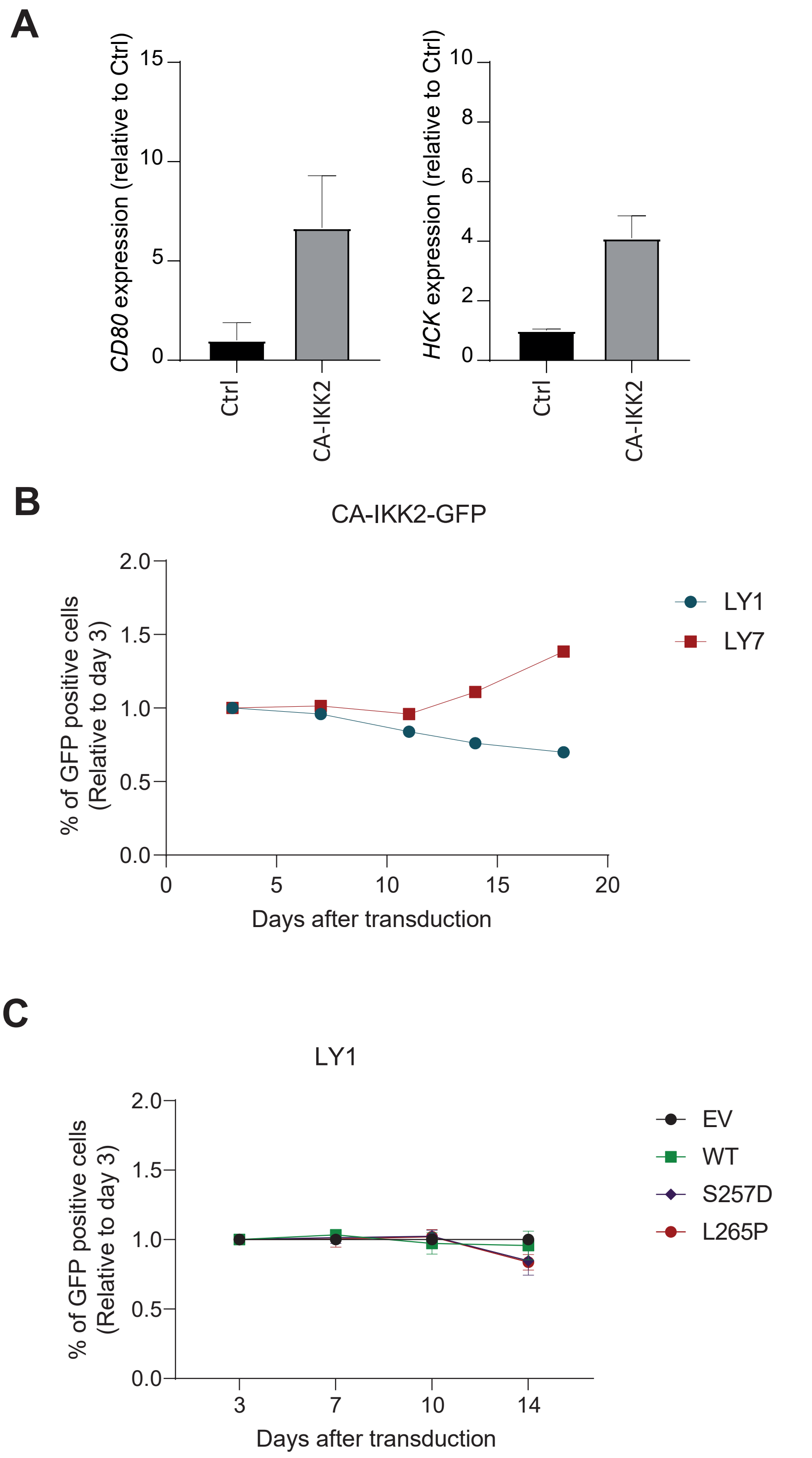

Supplemental Figure 2
(A) RT-qPCR analysis of CD80 and HCK expression in OCI-LY7 transduced with CA-IKK2-GFP. Cells were allowed to recover for 72 hours before RNA isolation. RPLP0 was used as an input control and data are normalized to the control expression levels. (B) Flow cytometric analysis of OCI-LY1 and OCI-LY7 cells transduced with a vector expressing CA-IKK2 and co-expressing GFP. The percentage of GFP positive cells was followed in time and plotted as the percentage of GFP+ cells, normalized to the value at day 3 following retroviral transduction. (C) Flow cytometric analysis of OCI-LY1 cells transduced with an empty vector (EV) or an expression vector for MYD88 (WT, S257D or L265P) co-expressing GFP. The percentage of GFP positive cells was followed in time and plotted as the percentage of GFP+ cells, normalized to the value at day 3 following retroviral transduction. The mean ± S.E.M. of two independent transductions is shown.

Supplemental Figure 3

A

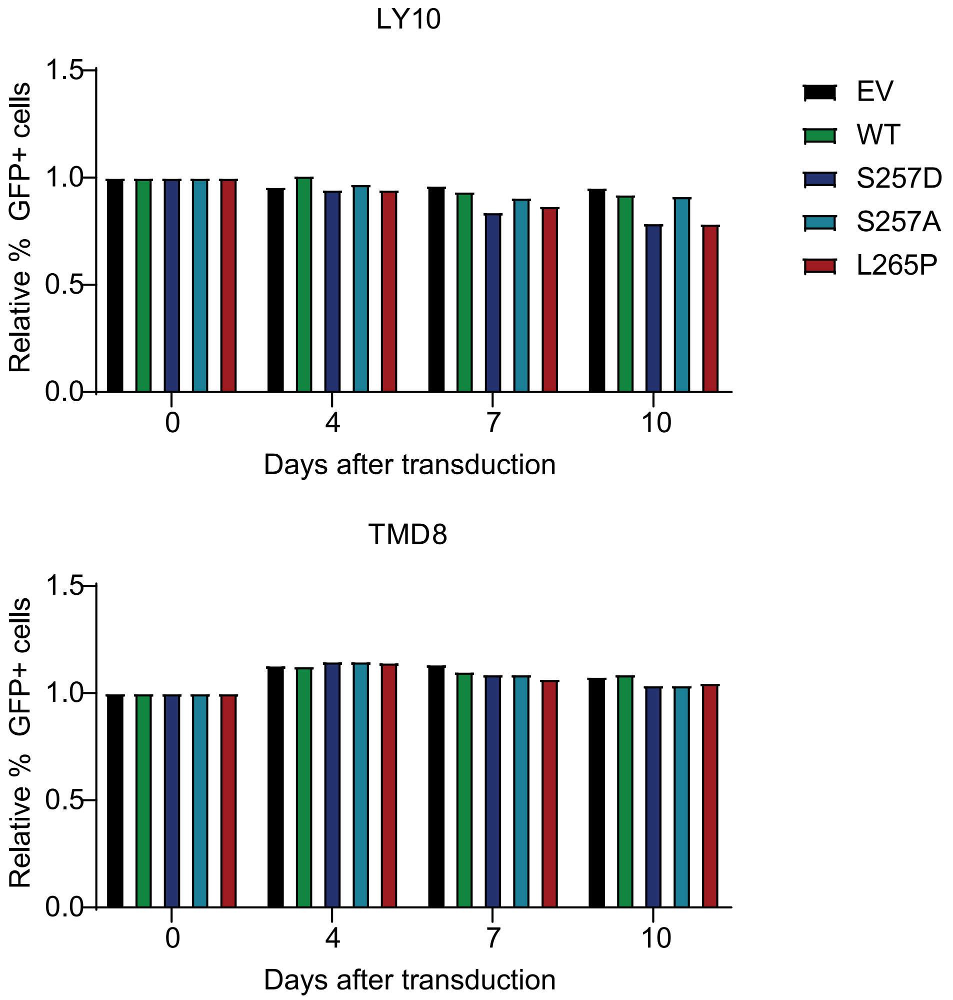

Supplemental Figure 3
(A) Flow cytometric analysis of OCI-LY10 and TMD8 cells transduced with an empty vector (EV) or an expression vector for MYD88 (WT, S257D, S257A or L265P) co-expressing GFP. The percentage of GFP positive cells was followed in time and plotted as the percentage of GFP+ cells (relative to day 0).
